# Special Issue “Halophilic Microorganisms”

**DOI:** 10.3390/microorganisms11030690

**Published:** 2023-03-08

**Authors:** Cristina Sánchez-Porro

**Affiliations:** Department of Microbiology and Parasitology, Faculty of Pharmacy, University of Sevilla, 41012 Sevilla, Spain; sanpor@us.es

Hypersaline environments are mainly represented by aquatic systems, such as solar salt ponds or natural salt lakes, as well as by the sediments of these hypersaline aquatic ecosystems and soils with high salt content [1]. Also constituting hypersaline environments are mines and salt deposits; the interiors of some desert plants; skins tanned with saline solutions; and a variety of brined, salted, or fermented products [2].

Halophiles are microorganisms adapted to live in these hypersaline environments and in other saline products. Most of them belong to the bacteria and archaea domains, although eukaryotes are also present (especially fungi), and their interest is of special relevance for their adaptation mechanisms to extreme conditions, the different molecules that they are able to produce (enzymes, polysaccharides, etc.), and for their biotechnological applications [3].

The isolation, study, and taxonomic characterization of halophilic microorganisms have allowed us to learn more about their heterogeneity, their metabolic and physiological diversity, and their ecological distribution and biodiversity. Culture-independent techniques, such as metagenomic and -omic studies, have been particularly interesting in recent years, providing valuable information regarding the diversity and biotechnological potential of these environments and the microorganisms that inhabit it [4].

This Special Issue collects seven original works related to halophilic microorganisms and/or their potential applications, as well as a review which includes an exhaustive compilation of the main antimicrobial compounds retrieved from microbial sources of various saltern environments.

The biodiversity in hypersaline environments has been studied by numerous researchers for years. Both classical culture techniques and metagenomic techniques have been used. The four research papers in this Special Issue are related to the study of biodiversity in different hypersaline environments [5,6,7,8].

Akpolat et al. [5] studied the prokaryotic community in 30 hypersaline environments located in different areas of Turkey using 16S rRNA amplicon sequencing (V6-V8 variable regions). The salinity of the samples ranged from 30% to 38%. The results indicated that most 16S rRNA gene sequences belonged to the domain *Archaea* (98.41%); in contrast, only 1.38% of the 16S rRNA gene sequences were related to the domain *Bacteria*. These results are in accordance with the characteristics of these environments, as their high salinity limits diversity, and the extremely halophilic archaea are the dominate population. At the genus level, most of the sequences were classified into the genus *Haloquadratum* (in the *Archaea* domain); with respect to *Bacteria,* most of them were related to the genus *Salinibacter*. This study also highlighted the detection of 16S rRNA gene sequences in great abundance. These were related to two uncultured microorganisms and belonged to the class *Halobacteria* (*Archaea),* therefore demonstrating the great effort that still remains to be made to isolate new microorganisms in a pure culture.

Another work included in this Special Issue, aimed at the search for hydrolytic enzymes in hypersaline environments in Romania, isolated 244 microorganisms in a pure culture, of which 74.6% were represented by bacteria, 9.0% were represented by archaea, and 16.4% were represented by fungi. The salinity of the five studied samples ranged from 11.3 g L^−1^ to more than 70 g L^−1^ (the detection limit of the instrument used). The low salinity of the samples explains why most of the isolates (57.8%) were categorized as halotolerant. Analysis of the partial 16S rRNA gene sequences of the prokaryotic isolates (fungal isolates were not taxonomically identified) revealed that the isolated bacterial belonged to three phyla: *Bacillota* (formerly *Firmicutes*), *Pseudomonadota* (formerly *Proteobacteria*), and *Actinomycetota* (formerly *Actinobacteria*), and the archaeal isolates fell within three orders of the class *Halobacteria* (*Halobacteriales, Haloferacales,* and *Natrialbales*) [6].

The third work related to biodiversity in these hypersaline environments was carried out by Duran-Viseras et al. [7]. In their study, they describe a new, slightly halophilic bacterium, *Marinobacterium ramblicola*, isolated from Ramba Salada (Murcia, Spain). This work exhaustively studied not only phylogenomic and phenotypic characteristics, but also performed a complete genome-based metabolic reconstruction of the new species. The researchers inferred a versatile energetic metabolism for this new taxon, and based on the genomic inspection, they suggested that the new species was able to biosynthetize or catabolize compatible solutes and to degrade several aromatic hydrocarbons. These characteristics, among others, allow this microorganism to live in hypersaline environments.

The latest work related to biodiversity in these environments also considers other factors. In hypersaline environments, there are often other characteristics besides salt that make them extreme environments for most organisms; one of these additional factors is the presence of heavy metals. An example of these features is a former uranium mining and milling site in Germany from which Harpke et al. [8] took soil, sediment, and water samples to study the adaptation of microbial communities to this extreme environment. They screened for isolates surviving the high salt and metal concentrations. The authors determinedthe biodiversity both through classical, culture-dependent studies and through culture-independent techniques using sequencing performed by by Illumina MiSeq platform. The 16S rRNA microbiome analysis showed a strong selection for bacterial phyla. *Pseudomonadota* (formerly *Proteobacteria*), *Chloroflexota* (formely *Chloroflexi*), and *Acidobacteriota* dominated, followed by *Actinomycetota* (formerly *Actinobacteria*) and *Bacteroidota*. Surprisingly, no sequence related to the archaeal domain was detected. With respect to the fungal microbiome, *Ascomycota*, *Mortierellomycota*, and *Chytridiomycota* were dominant. From the culture-dependent studies, 181 pure cultures were obtained, and isolates members of the phyla *Bacillota* (*Bacillaceae* family) and *Actinomycetota* were dominated. In addition, halophilic species belonging to the genera *Halobacillus* and *Halomonas* were present. The fungi were represented by members of the phylum *Ascomycota*. Furthermore, the resistance mechanisms prevalent in this extreme environment were checked, and biomineralization of Cs- and Sr-struvite was identified to be present in most of the strains. These mechanisms may be of specific interest for bioremediation approaches.

Hypersaline environments are environments in which the microorganisms living there must develop specific mechanisms to be able to live in those conditions. A research article included in this Special Issue deals with these mechanisms. The work carried out by Matarredonda et al. [9] studies, in detail, the adaptation mechanisms of the arquea *Haloferax mediterranei* to conditions of high salinity, pH, temperature, oxidative stress, and heavy metals. *Haloferax mediterranei* grows optimally at 20% (*w*/*v*) salt, pH 7.25, and 42 °C. It also can tolerate Li^+^, Co^2+^, As^5+^, and Ni^2+^ at different concentrations in defined media. All these characteristics allow this microorganism to be considered as a microorganism with a polyextremophilic character, useful for developing new biotechnological applications.

Halophilic microorganisms can also be sources of enzymes of great interest and with possible applications in industry, since they are enzymes that may possibly be active across a broad range of salt salinities, and this property is of special importance in industries such as food processing, leather, textiles, and detergents, as well as in waste treatment applications. Two works in this Special Issue are related to the characterization of enzymes. One of them is the aforementioned work by Ruginescu et al. [6], in which the authors, apart from the biodiversity study which they carried out, performed screening of the isolates to detect enzymatic activities such as proteases, lipases, amylases, xylanases, cellulases, and pectinases. Of the 244 isolates, 156 produced single or combined hydrolytic activities. The other study is the work carried out by Chung et al. [10]. They isolated a fungal strain from a hypersaline saltern in Korea, and it was identified as *Aspergillus reticulatus* strain SK-1. It produces an extracellular alkaline serine protease with activity over a broad range of NaCl concentrations, making it very interesting for its widespread applicability in various industries.

The last article in this Special Issue is concerned with whether the life forms that we know would be able to survive under extreme conditions. They studied the effect of a high concentration of sodium sulfate on the cell growth, death, morphology, cell division, and gene expression of *Escherichia coli*. These studies are very interesting for the purposes of investigating the potential habitability of other planetary bodies of astrobiological importance [11].

Finally, this Special Issue also includes an interesting review of the potential interest shown in halophilic microorganisms as source sof new antimicrobial compounds. Antibiotic resistance is a growing problem, and the search for new antibiotics has become a priority. This work shows how halophilic microorganisms can be a potential source of antimicrobials, with species of the genus *Bacillus* and members of the Phylum *Actinomycetota* being the most promising. Other halophilic bacterial species and halophilic microalgae are included, as well as the use of halo-microbial derived products such as pigments, biosurfactants, and exopolysaccharides, in the review of possible antimicrobial compounds [12].

In summary, the seven original articles and the review published in this Special Issue, “Halophilic Microorganisms”, are an excellent representation of the research in this field, showing the importance of biodiversity and industrial applications as well as the adaptation mechanisms of these microorganisms.

## Data Availability

Not applicable.
